# Changing Perception of Avian Influenza Risk, Hong Kong, 2006–2010

**DOI:** 10.3201/eid1712.110298

**Published:** 2011-12

**Authors:** Qiuyan Liao, Benjamin J. Cowling, Wing Tak Lam, Richard Fielding

**Affiliations:** University of Hong Kong, Hong Kong, People’s Republic of China

**Keywords:** H5N1, influenza, risk perception, behavior, change, viruses, Hong Kong

**To the Editor**: Since 1997, routine surveillance has demonstrated periodic reemergence of influenza A (H5N1) viruses (avian influenza) in retail markets in Hong Kong, People’s Republic of China (*1*,*2*), leading to stepped implementation (progressively implementing more measures over time) of measures to reduce human exposure to influenza subtype H5N1. From 2006 through November 2008, progressive importation and farm restrictions and curtailed retail capacity cut Hong’s live poultry supply in half, from 40,000 to <20,000 chickens daily (*3*,*4*).

To determine whether the decline in the Hong Kong live poultry supply was paralleled by declines in avian influenza risk perceptions and protective hygiene behavior, we conducted a telephone survey. During December 2005–March 2006, we recruited 1,760 adults >17 years of age. We randomly called households and then interviewed 1 adult (randomly selected by Kish grid) within each household (*5**,**6*). Ordering by age and starting from the oldest eligible member in the household, 1 selected member was then invited to participate in the survey. Of 1,613 (92%) respondents consenting to follow-up survey, 680 (42%) were resurveyed during July–August 2010. The same items were used in both surveys to measure avian influenza risk perceptions, personal live poultry exposures, and hygiene practices.

Overall, 461 (68%) respondents completed the initial (2006) and follow-up (2010) surveys. Compared with respondents lost to follow-up, these 461 respondents were more likely to be female, slightly older, and married; they were comparable with the general population (*7*), except more respondents were older (data not shown).

Respondents perceived that their likelihood of contracting avian influenza was the same in 2010 as in 2006, but they reported worrying less about contracting avian influenza and risks from buying live poultry in 2010 than in 2006 (Table). When categorized into “unchanged,” “increasing,” and “declining” in 2010 relative to 2006, these groups were comparable demographically, except younger respondents more often perceived declining likelihood of contracting avian influenza (odds ratio [OR] 2.30, 95% confidence interval [CI] 1.25–4.24 for those 18–34 years of age); declining worry about contracting avian influenza (OR 2.01, 95% CI 1.10–3.66 for those 18–34 years of age; OR 1.87, 95% CI 1.13–3.09 for those 35–54 years of age), and declining risk from buying live poultry (OR 2.31, 95% CI 1.33–4.01 for those 35–54 years of age); respondents who had completed secondary education were more likely to report declining worry about contracting avian influenza (OR 1.90, 95% CI 1.09–3.31).

**Table:** Changes in perception of risk for avian influenza, live poultry exposure, and hygiene practices, Hong Kong, 2006­­­­–2010*

Perception or practice	2006 survey	2010 survey	Differences, p value
Risk perception			
Perceived likelihood (likely/very likely/certain)	18	14	0.201†
Worry (worry a bit/a lot/all the time)	26	21	<0.001†
Perceived risk from buying live poultry (somewhat agree/agree/strongly agree)	42	31	<0.001†
Live poultry exposure			
Buying live poultry (yes)	73	41	0.009‡
Frequency of purchase (at least monthly or more frequently)	50	15	<0.001†
Purchase rate among buyers (chickens/household/y )	14.4	11.4	
Frequency of touch among buyers (sometimes/usually/always)	8	6	0.326§
Averaged touch rate among buyers	0.05	0.05	
Averaged exposure among buyers (exposure/household/y)	0.72	0.57	0.011†
Personal hygiene practices			
Frequency of washing hands (at least hourly)	49	44	0.023†
Covering mouth when sneezing or coughing (usually/always)	91	85	0.003†
Washing hands after sneezing, coughing, or touching nose (usually/always)	72	75	0.027†
Using liquid soap when washing hands (usually/always)	63	74	<0.001†
Using serving utensils when dining with others (usually/always)	40	62	<0.001†

*Total population surveyed = 461. Values are % unless otherwise stated. †Wilcoxon signed rank test. ‡McNemar test. §χ^2^ test.

The percentage of respondents who reported household buying of live poultry declined from 73% in 2006 to 41% in 2010 (Table); 22% of nonbuying households in 2006 were again buying in 2010, and 52% of those buying in 2006 had stopped buying in 2010. After adjustment for demographics, perceived increased risk from buying was associated with not buying live poultry in 2010 (OR 0.34, 95% CI 0.19–0.60).

In contrast, rates of touching poultry during buying (5%) were unchanged (Table). Using a standardized estimate (*5*), we determined that purchasing households bought on average 11.4 live chickens/household/year in 2010 versus 14.4 in 2006 (Table). Purchase rate × touch rate gave an estimated average of 0.57 exposures/household/year in 2010, a 21% decline from 0.72 exposures/household/year in 2006 (p = 0.011) (Table).

Substantial improvement was noted for most personal hygiene practices, except frequencies for daily handwashing and covering the mouth when sneezing or coughing were each lower in 2010 than in 2006 (Table). Changed hygiene practices were independent of demographic factors except that male respondents more often reported less covering of the mouth when sneezing or coughing (OR 1.60, 95% CI 1.00–2.56) and less use of liquid soap for handwashing (OR 1.64, 95% CI 1.04–2.60); immigrants were more likely to have reduced daily handwashing frequency (OR 1.58, 95% CI 1.04–2.41). Only perceived declining worry about contracting avian influenza was significantly associated with declining frequency of handwashing after sneezing, coughing, or touching the nose (OR 1.61, 95% CI 1.04–2.47).

The 21% decline in exposure from less buying, but not touching, of live poultry suggests that limiting poultry availability, but not health education efforts, was responsible. Perceptions of avian influenza risk and worry also mostly declined, as did frequency of some personal hygiene practices, including handwashing, particularly among younger male and immigrant respondents. Although our previous studies suggest that public health education might have contributed to an ≈43% reduction in rate of touching when buying live poultry in Hong Kong from 2004 to 2006 (*5**,**6*), the prolonged warning that a future pandemic is likely to be sparked by influenza A (H5N1) viruses is likely to cause pandemic fatigue in the public and therefore would not change their perception of avian influenza risk and associated protective behavior. As exposure risk has declined (as a result of government policy), so has perceived infection risk also declined, paradoxically increasing population vulnerability to other influenza viruses through reductions in preventive hygiene behavior.
